# Caregiver Perceptions of Music Therapy for Children Hospitalized for a Blood and Marrow Transplant: An Interpretivist Investigation

**DOI:** 10.1177/2164956118788853

**Published:** 2018-07-18

**Authors:** Greta J Yates, Nicole B Beckmann, Megan E Voss, Maureen R Anderson, Michael J Silverman

**Affiliations:** 1 Masonic Children’s Hospital, University of Minnesota, Minneapolis, Minnesota; 2 Earl E. Bakken Center for Spirituality and Healing, University of Minnesota, Minneapolis, Minnesota; 3 School of Music, University of Minnesota, Minneapolis, Minnesota

**Keywords:** pediatrics, blood and marrow transplant, caregivers, experiences, integrative, music therapy

## Abstract

**Background:**

Despite pharmacological and psychosocial support, pediatric blood and marrow transplant (BMT) recipients typically experience heightened levels of somatic and psychological distress while undergoing transplant. Although clinicians have used psychosocial interventions to target distress, there are gaps in the literature concerning music therapy for children recovering from BMT. This is especially the case among younger children and those affected by rare genetic or metabolic disorders.

**Objective:**

As caregivers are the primary decision makers for minor children, there is a need to understand their preferences and values as this constitutes a component of evidence-informed practice. Therefore, the purpose of this interpretivist investigation was to explore primary caregivers’ perspectives and experiences with music therapy for their children during hospitalization for BMT.

**Methods:**

The researchers conducted semistructured phone interviews with 15 primary caregivers of children who had received music therapy services while hospitalized for BMT. Member checking was used to ensure accuracy of the transcripts and experiences, while trustworthiness was used to verify themes.

**Results:**

Fifteen caregivers of 14 pediatric BMT recipients were interviewed by phone. The following four themes emerged: (1) music therapy motivated patients to physically engage their bodies despite symptoms; (2) music therapy elevated patients’ moods; (3) caregivers benefited from seeing their child engaged in music therapy; and (4) caregivers were appreciative for the opportunity and requested additional music therapy services.

**Conclusions:**

Caregivers perceived music therapy to be a positive and beneficial experience for pediatric patients while hospitalized for BMT. Implications for clinical practice, limitations, and suggestions for future research are provided.

## Introduction

Pediatric blood and marrow transplant (BMT) recipients experience significant emotional and somatic distress related to transplant, which may impact functioning for years following BMT.^1–5^ Nonpharmacological interventions targeting distress following BMT can include humor therapy,^1,6,7^ massage therapy,^1,6,7^ and music therapy.^8^ Due to the high involvement levels of caregivers of pediatric BMT recipients, caregivers often function as advocates and decisions makers for their children, and there is a need to understand their perspectives and experiences with nonpharmacological interventions such as music therapy. In understanding caregiver preferences and experiences, music therapists can be better equipped to advocate, meet the needs, and provide better quality services to children and their caregivers. As patient values and preferences constitute an integral component of evidence-informed practice, caregivers can represent an extension of their children as the primary decision maker and may provide valuable insights into their children’s values and experiences. Therefore, the purpose of this interpretivist investigation was to explore primary caregivers’ perspectives and experiences with music therapy for their children during hospitalization for BMT.

## Literature Review

In 2014, there were over 21,000 pediatric and adult BMTs in the United States.^9^ Side effects of treatment and the standard confinement to a HEPA air filtration room to prevent infection can make BMT a stressful event for pediatric BMT patients and their caregivers.^1–4^ Although some BMTs are curative treatments, others aim to halt disease progression, decrease symptom burden, and enhance quality of life. Depending on the diagnosis, patients will receive an autologous transplant of their own cells or an allogenic transplant harvested from umbilical cord blood or a donor following 7 to 10 days of preparative conditioning regimen of myeloablative chemotherapy and/or radiation specific to their disease.^10^ Common adverse effects of transplant include mucositis, pain, nausea and vomiting, insomnia, physical deconditioning, infection, and bleeding.^11^ Symptom burden and side effects of treatment range in intensity depending on the type of transplant and the preparative conditioning regimen. Allogeneic BMTs typically result in greater symptom burden and longer length of hospital stay. Symptoms typically begin to alleviate following white blood cell count recovery, referred to as engraftment. BMT recipients are hospitalized for preparative conditioning and are not discharged until white blood cell recovery is achieved, typically 14 to 28 days after transplant. Children are hospitalized for approximately 4 to 6 weeks for the initial BMT stay. BMT-related complications within the first 100 days are common and may result in readmissions and frequent clinic appointments.^10^

BMT hospitalization and the standard confinement to a HEPA air filtration room to prevent infection often cause increased stress and anxiety to pediatric patients and their caregivers that peaks during preparative conditioning and often does not return to baseline until 6 months posttransplant.^1–5,12^ However, many pediatric BMT recipients report continued anxiety and feelings of vulnerability during survivorship.^5^ Compromised emotional functioning, high levels of worry, reduced communication during the most acute phase of transplant, more intensive treatments, and the child’s cognitive, behavioral, and social functioning predicted lower quality of life ratings.^4,13,14^ Pediatric oncology programs may offer a variety of nonpharmacological interventions, including acupuncture, aromatherapy, massage, music therapy, and supplements, as part of their standard of care.^15^ The contemporary literature base suggests that nonpharmacological interventions, such as music therapy, may lessen emotional and somatic distress post BMT.^1,2,8,16^

Music therapy is the “use of music interventions to accomplish individualized goals within a therapeutic relationship by a credentialed professional who has completed an approved music therapy program.”^17^ Among the 7642 board-certified music therapists represented globally in 2017, it is estimated that approximately 10% of therapists are employed within pediatric hospitals.^18^ Music therapy is a professional field consisting of a plethora interventions based on the individual assessment and strengths of each patient. BMT music therapists emphasize the importance of rapport and flexibility through various interventions to promote structure, autonomy, and engagement to provide an opportunity for expression that may result in enhanced coping skills and recovery.^8,16,19–21^ As the number of music therapists grows and services expand to broader patient populations, there is a need for ongoing research regarding if and how music therapy can enhance quality of care and delivery of clinical services.

Music therapy can serve a variety of psychosocial functions depending on the context and the individual’s needs. For example, music therapists can help an individual work through conflict, lead to insightful reflection of an event, or simply invoke pleasure.^22^ Studies on patients with cancer have shown that music therapy interventions, including listening to live preferred music, have physiological benefits of decreasing pain, heart rate, respiratory rate, and blood pressure.^23,24^ In child-focused interventions including songwriting, improvisation, singing, and instrument play, music therapy increased child attention, affect, and autonomy as well as social integration and overall self-concept.^8,25,26^ Within a BMT population with cancer, adolescents and young adults (AYAs) participated in songwriting lyrics and melody and then created a music video of their composed song set to preferred photos. Participants experienced improved courageous coping and parents found enhanced understanding of the AYA’s emotions and connectedness with the child through listening to the composed lyrics and observing their child’s responses.^8,21^

Although music therapy can be beneficial for AYAs undergoing BMT for cancer diagnoses, there is a lack of research pertaining to music therapy with children under the age of 12 or among children undergoing BMT for rare genetic or metabolic disorders. As the indications for BMT continue to grow and BMT is performed more routinely in younger children, there is a need to understand the benefits of music therapy among these diverse populations. With a lack of clinical research, there is a need to explore patient and caregiver values and preferences in order to provide the best evidence-informed practice. Caregivers are the advocates and decision makers for their minor child, therefore they may be considered an extension of the child and provide valuable insights in to their preferences. The purpose of this interpretivist investigation was to explore primary caregivers’ perspectives and experiences with music therapy for their children during hospitalization for BMT. The guiding research question was as follows: What are caregivers’ perspectives and experiences with music therapy for their child hospitalized for BMT?

## Method

### Program Description

The setting was a 24-bed BMT unit at an urban pediatric hospital in the Midwestern region of the United States. On average, 80 children receive BMT a year at this institution. Approximately 50% of patients are transplanted due to a malignancy and 50% of patients have rare genetic or metabolic disorders including diseases such as epidermolysis bullosa, Hurler syndrome, and adrenoleukodystrophy. Approximately 75% of BMTs at this institution are allogeneic. In 2017, 54% of pediatric BMT recipients at this hospital were aged 5 years or younger.

The integrative health program at this institution was established in 2014 to help alleviate symptom burden following BMT and to enhance patient and family reslience and capacity to cope. Services provided by the integrative health team include massage, music therapy from a board-certified music therapist, healing touch/Reiki, acupressure, mind/body skills, yoga, and aromatherapy. Inpatient music therapy began in 2016 with exclusive availability to pediatric BMT recipients 10 to 28 hours per week. All patients are offered integrative therapy and music therapy upon their admission to the unit. Approximately 70% of all admitted BMT patients elected to receive music therapy services in 2017.

Music therapy sessions typically range from 15 to 45 minutes in duration, and children receive 2 sessions per week until discharge. The principal investivator (PI) of this study is a board-certified, master’s level practitioner with 3½ years of experience who primarily utilizes cognitive behavioral music therapy approaches. Common music therapy interventions implemented on the unit include active music engagement through instrument play, music for physical engagement, music for relaxation, instrument lessons, and musical books.

### Research Participants and Recruitment

Caregivers of children who received music therapy services during the initial BMT hospital stay were invited to participate in the study prior to hospital discharge. Caregivers were included if they were a primary and legal caregiver of a child receiving BMT who participated in music therapy services and the ability to read, write, and speak in English. If more than one of the child’s caregivers met inclusion criteria, all were approached for participation. Non–English-speaking caregivers and caregivers of children on the BMT unit who had not received a transplant were excluded. Thus, purposive sampling was used in an attempt to understand the perceptions and experiences of the research participants.

### Procedure

Prior to recruitment, this project was approved by the university and hospital shared institutional review board (1609S95284) as well as the leadership in the BMT program. The PI obtained written consent at the time of recruitment in addition to e-mail and telephone contact information for each participant. A researcher employed as a nurse practitioner on the BMT unit called the caregiver and conducted individual semistructured telephone interviews. If the caregiver was unable to be contacted after 2 attempts, the participant was considered withdrawn. Interviews were audio recorded, transcribed, and read by the PI who removed redundancies. Transcripts were then sent to participants by e-mail for member checking. Participants did not receive payment for their participation.

### Semistructured Interview

A semistructured interview composed of 5 open-ended questions was initially developed by the PI and modified and refined by the research team to ensure the questions were nonleading and appropriate for the research question. The questions asked caregivers to reflect generally on their experiences during their child’s BMT hospital stay. The interview then progressed to questions central to the caregiver’s and the child’s experiences with music therapy during the initial BMT hospitalization.

### Qualitative Analysis and Trustworthiness

Due to the exploratory nature of the study, the researchers used thematic analysis as a guiding framework to understand caregivers’ experiences and perceptions of music therapy for their child undergoing BMT. Braun and Clarke’s^27^ 6 phases of thematic analysis was used for data analysis and entails: (1) familiarization with the data; (2) generation of initial codes; (3) searching for themes; (4) reviewing themes; (5) defining and naming themes; and (6) producing the report.

During analysis, the PI highlighted and coded participants’ quotes in the margins of the transcript. The PI then copied and pasted quotes and codes into separate documents and organized the quotes by their codes. Similar codes were then grouped together allowing the opportunity to compare and contrast codes between participants. After review, revision, and discussion, themes emerged from the related code categories. To ensure trustworthiness, the PI met with all researchers affiliated with the project and discussed data, reviewed codes, resolved discrepancies, clarified themes, and verified that quotes pulled were grounded in the themes. Members of the research team provided feedback to the PI that was then integrated into the development and refinement of the themes. The data were then triangulated with the existing literature.

## Results

Nineteen caregivers were approached for participation and provided informed consent. Two caregivers never responded to the researcher’s voicemails and 2 caregivers were not approached following the unanticipated death of their child. Fourteen participants in the study were parents of the child and 1 participant was a grandmother. Two participants were parents of the same child. Ten of the patients were female. Patients ranged in age from 1 to 16 years. Nine patients were under the age of 5. Fifty percent of patients had a rare genetic or metabolic diagnosis and 13 underwent allogeneic transplant. Patients ranged from 18 to 130 days post-BMT at time of interview, averaging 62 days post-BMT. This sample is representative of the unit’s demographics. Interviews lasted from 3 to 15 minutes.

**Figure 1.**
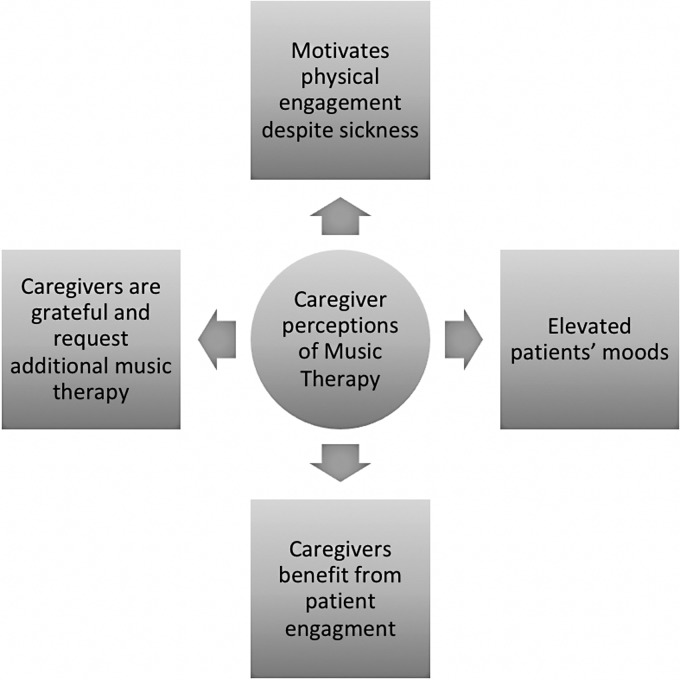
Identified Themes.

Four themes were identified via the thematic analysis (Figure 1). Codes are depicted to support themes and enhance transparency. Representative quotes are included to provide contextual support for the themes.

## Theme 1: Music Therapy Motivated Patients to Physically Engage Their Bodies Despite Their Symptoms

### Codes: Sick, Motivation, Movement, Physical

Caregivers noted that their child experienced high levels of symptom burden during the acute phase of transplant. Due to the high levels of somatic distress and necessary medication, patients also experienced increased fatigue and became less engaged in physical activities. Some patients refused to get out of bed or sit up in bed and were at risk of physical deconditioning. However, music therapy motivated patients to engage physically in an activity despite their symptoms. Even when patients refused other services or engaged less with family, they often continued to be participatory in music therapy sessions. Although music therapy interventions are sometimes more passive during this phase, active interventions may be modified to augment the likelihood of participation. For example, a patient may sit at the edge of the bed instead of playing on floor mat or play lighter instruments to promote physical engagement.

The days she felt pretty rotten, even if she was lying in bed trying to sleep or rest quietly or whatever, and we weren’t able to get her to be active. When Greta would come through the door, she would get up and participate in [music] therapy. So, that helped. It helped to get her motivated to sit up and be active... [On days without music therapy] it was difficult to get her up and motivated. She would, if physical therapy came in and made her, she would, but very reluctantly. Her mood just seemed, maybe a little bit relaxed on music therapy days. (Participant 1)

## Theme 2: Music Therapy Elevated Patients’ Moods

### Codes: Relaxation, Calm, Comfort, Mood, Spirits, Looked Forward to

Participants noted that being confined to a hospital room can be isolating, monotonous, and negatively impact their child’s moods. Not only does music therapy motivate a child to engage their bodies physically during times of increased symptom burden, but caregivers noted that it also elevated their child’s mood. Music therapy provided patients and their families the opportunity to engage in a more typical childhood experience that was perceived as enjoyable despite being hospitalized. Caregivers described music therapy as a “highlight” of the hospitalization that the patients looked forward to despite symptoms. Music therapy provided the opportunity to “reset” a child’s day and redirect them from pain, nausea, fatigue, or unpleasant procedures by creating a calm and engaging environment.

I think it was always, no matter how she was feeling, it was always a little bit of an escape for her... It got her to a different place I think. It got her to someplace else. It snapped her out of the funk she was in or whatever... A lot of times it felt like a reset button. It was changing course of our day... It would just shift her mood in to a different direction. (Participant 6)

## Theme 3: Caregivers Benefited From Seeing Their Child Engaged in Music Therapy

### Codes: Relief, Beneficial, Peaceful, Happy, Helpful

Caregivers described BMT as “stressful, scary, and hard.” When watching their child engage in music therapy, caregivers noted the opportunity to see their child be a “different kid.” Some children had never been hospitalized for an extended period of time before or were not as ill with previous treatments. Caregivers experienced distress when they observed their child sick and in distress as they “couldn’t fix it.” Seeing their child happy, participatory, and engaged in music therapy provided relief, peace, and comfort to caregivers amidst the misery of transplant.

When [she] was smiling, it forced me. Her mom and I both smiled. Her enjoying it made us feel happy to see her have some enjoyment for that time and absolutely not be thinking of her sickness. (Participant 3)

## Theme 4: Caregivers Were Appreciative for the Opportunity and Requested Additional Music Therapy Services

### Codes: Grateful, Thankful, Complimentary, Availability, Program

All caregivers were appreciative of the opportunity for their child to participate in music therapy throughout the transplant process and spoke highly of their experiences. On average, patients received music therapy 2 times a week while hospitalized. BMT requires a long isolating hospital stay, which precludes participation in hospital-wide activities for patients. Therefore, participants noted that the need for stimulation and engagement is imperative. As such, caregivers requested additional music therapy services for their child, not only due to isolation, but also for the child’s enjoyment, quality of life, and level of engagement in music therapy.

Now that we’re back in our apartment, we sing. We dance. It made singing fun again... It brought music back to us. We will forever be grateful that she came to see us... Sometimes, it’s hard to know, I would have even said, “She could come in every day.” I know that’s not fair. I know there’s other little boys and girls and there is only one of her. It’s hard, but it would have been nice to see her more. She was just so happy when she was there. (Participant 12)

## Discussion

The implementation of nonpharmacological interventions, such as music therapy, in acute inpatient medical settings can result in a patient-centered model that may improve patient satisfaction.^28^ Therefore, the purpose of this interpretivist investigation was to explore primary caregivers’ perspectives and experiences with music therapy for their children during hospitalization for BMT. Fifteen caregivers of 14 patients participated in semistructured phone interviews to understand their perceptions and experiences with music therapy for their child undergoing BMT. Member checking and trustworthiness were incorporated. Emerging themes included are the following: (1) music therapy motivates patients to physically engage their bodies even when feeling sick; (2) music therapy elevated patients’ moods; (3) caregivers benefit from seeing their child engaged in music therapy; and (4) caregivers are thankful for the opportunity but request additional music therapy services.

Consistent with previous work with AYAs after BMT,^8,21^ this study found that there are mutual caregiver and child benefits from music therapy after BMT. This work suggests that music therapy can benefit BMT patients—and their caregivers—regardless of age or underlying diagnosis. Not only does music therapy improve mood and combat feelings of isolation, music therapy was integral in promoting physical activity. As patients experience a decrease in strength and exercise capacity following BMT,^29^ music therapy may be advantageous in helping prevent further deconditioning. Some caregivers commented on the outcomes of music therapy including decreased heart rate, decreased pain, and increased relaxation. Quantitative findings from the existing literature concerning the impact of music therapy on decreasing pain, heart rate, respiratory rate, and blood pressure are congruent.^23,24^

Music therapy also provided patients and their caregivers the normalizing opportunity to engage in a typical childhood experience and something to look forward to when the patient was immunocompromised and isolated in their room. This finding is consistent with previous music therapy literature where children experienced increased autonomy and improved social integration and affect.^8,25,26^ Music therapy also provided the opportunity for caregiver respite and self-care, so caregivers were not always present during the session. Regardless of their participation or presence, all caregivers commented that music therapy enhanced their experience during hospitalization even though music therapy interventions were directed at their child.

### Implications for Clinical Practice

Results suggested that music therapy was perceived to be a beneficial and positive experience for pediatric patients and their caregivers while hospitalized after BMT. As BMT patients are typically hospitalized for longer durations, unable to participate in hospital wide services due to the standard confinement to prevent infection, and experience immense symptom burden, it is reasonable to consider that BMT patients should receive frequent nonpharmacological interventions. Caregivers recommended scheduling music therapy services more regularly to further enhance the hospital stay and promote the child’s physical activity and mood. Participants spoke positively concerning the music therapist’s flexibility and ability to work with their child throughout BMT, regardless of their child’s ability to engage in sessions or severity of symptoms. The importance of a music therapist’s flexibility and scheduling has also been emphasized by other music therapists working within pediatric oncology and BMT.^19–21^

### Limitations

Limitations of this study included the interviews being conducted by phone and occurring while the caregiver was home with their child. Caring for BMT patients after discharge is stressful and time consuming and requires many clinic appointments a week. Caregivers were not always able to talk at length with the interviewer due to the extensive needs of their child. Interviews conducted in person without the presence of the child may have resulted in longer and deeper conversations with caregivers. In addition, male caregivers are underrepresented in the sample or research participants. Finally, results of this interpretivist study are limited by the makeup of the research team, which was composed of experts within pediatric BMT and integrative health. Thus, it would be impossible to separate these clinical experiences from the researchers’ way of knowing, interpreting, and understanding the data.

### Suggestions for Future Research

Given the lack of research with this population, caregivers’ perceptions of music therapy and its benefits are encouraging about its effects on patients and their caregivers throughout their transplant. This study supports the continued use of music therapy with this population and pursuit of expanding opportunities for patients and families throughout BMT recovery. Research exploring pediatric patients’ perceptions and the specific effects of music therapy interventions, especially on symptoms and psychosocial well-being, is warranted. Future work should attempt to measure the benefits of music therapy from the perspective of children less than 12 years of age and further explore the benefits of music therapy on physical activity and conditioning among children after BMT. Related to the results of the current investigation, additional research is needed to understand the impact of music therapy on physical and developmental deconditioning that is experienced after a child’s BMT. Objectivist research with randomization, control groups, established psychometric instruments to measure psychologic distress and symptom burden, and follow-up to determine potential maintenance of treatment gains is also necessary to gain a more holistic understanding of how music therapy might impact BMT patients and caregivers.

## Conclusion

The purpose of this interpretivist investigation was to explore primary caregivers’ perspectives and experiences with music therapy for their children during hospitalization for BMT. This study adds to the literature base and indicates that music therapy can be a beneficial psychosocial treatment modality for all pediatric BMT recipients, regardless of age or diagnoses. This study highlights the importance of music therapy in minimizing the effects of isolation, negative mood, decreased activity, and enhancing normalization of childhood experiences during BMT recovery. Music therapy is valued by caregivers to help mitigate the physical challenges of BMT and promote both the caregiver and child’s psychosocial well-being.
